# Antifungal Exposure and Resistance Development: Defining Minimal Selective Antifungal Concentrations and Testing Methodologies

**DOI:** 10.3389/ffunb.2022.918717

**Published:** 2022-06-13

**Authors:** Emily M. Stevenson, William H. Gaze, Neil A. R. Gow, Alwyn Hart, Wiebke Schmidt, Jane Usher, Adilia Warris, Helen Wilkinson, Aimee K. Murray

**Affiliations:** ^1^ European Centre for Environment and Human Health, University of Exeter Medical School, Cornwall, United Kingdom; ^2^ Environment and Sustainability Institute, University of Exeter Medical School, Cornwall, United Kingdom; ^3^ Medical Research Council Centre for Medical Mycology, University of Exeter, Exeter, United Kingdom; ^4^ Chief Scientist’s Group, Environment Agency, Horizon House, Bristol, England, United Kingdom

**Keywords:** antifungal resistance, antifungals, antimicrobial resistance, experimental evolution, selection, minimal selective concentration, fungi

## Abstract

This scoping review aims to summarise the current understanding of selection for antifungal resistance (AFR) and to compare and contrast this with selection for antibacterial resistance, which has received more research attention. AFR is an emerging global threat to human health, associated with high mortality rates, absence of effective surveillance systems and with few alternative treatment options available. Clinical AFR is well documented, with additional settings increasingly being recognised to play a role in the evolution and spread of AFR. The environment, for example, harbours diverse fungal communities that are regularly exposed to antifungal micropollutants, potentially increasing AFR selection risk. The direct application of effect concentrations of azole fungicides to agricultural crops and the incomplete removal of pharmaceutical antifungals in wastewater treatment systems are of particular concern. Currently, environmental risk assessment (ERA) guidelines do not require assessment of antifungal agents in terms of their ability to drive AFR development, and there are no established experimental tools to determine antifungal selective concentrations. Without data to interpret the selective risk of antifungals, our ability to effectively inform safe environmental thresholds is severely limited. In this review, potential methods to generate antifungal selective concentration data are proposed, informed by approaches used to determine antibacterial minimal selective concentrations. Such data can be considered in the development of regulatory guidelines that aim to reduce selection for AFR.

## Introduction

Antimicrobials, including antibacterial, antiviral, antifungal and antiparasitic agents, underpin modern medical practice and extensive agriculture (O’Neill, 2015; O'Neill, 2016; Fisher et al., 2018; Murray et al., 2018). However, the efficacy of antimicrobial drug therapy is increasingly challenged by the emergence and spread of antimicrobial resistance (AMR). AMR microorganisms, including bacteria, viruses, fungi, and parasites have the ‘ability to multiply or persist in the presence of an increased level of an antimicrobial agent’ (Ashbolt et al., 2013). Owing to drug-resistant infections of clinical importance, AMR research is well represented in the literature with respect to antibacterial resistance (ABR). In comparison, antifungal resistance (AFR) has been less well studied.

Fungi are a diverse group of eukaryotes, ranging from single-celled yeasts to complex, multicellular moulds (More et al., 2010). Numerous fungal species are responsible for invasive infections, with *Candida* spp. (yeasts) and *Aspergillus* spp. (moulds) being the major human fungal pathogens (Hill et al., 2015; Gow and Yadav, 2017). Collectively, invasive fungal infections cause more annual deaths than malaria or tuberculosis (Barnes et al., 2014; Van Dijck et al., 2018). Importantly, fungal communities are adaptable and may evolve resistance following antifungal exposure (Odds et al., 2003; Anderson, 2005; Perlin et al., 2017; Ksiezopolska and Gabaldon, 2018; Revie et al., 2018), making infections more difficult to treat.

As a consequence of the current COVID-19 pandemic, the urgency to tackle and mitigate AFR has increased due to critically ill COVID-19 patients suffering drug-resistant, invasive fungal infections. For instance, COVID-19 patients in India are reported to have suffered multidrug-resistant *Candida auris* infections, with high case-fatality rates (Chowdhary et al., 2020). Cases of COVID-19 associated aspergillosis and, the less common but serious infection, mucormycosis have also been reported (Singh et al., 2021). This exemplifies the importance of monitoring AFR and improving our understanding of the mechanisms behind the evolution of AFR across all ‘One Health’ sectors. The One Health approach to tackling AMR is inclusive of human, environment and animal health (Chowdhary and Meis, 2018). Previous research (Brown et al., 2012) highlights the threat of AFR in the clinic and the risk of AFR evolution due to the agricultural use of azoles, but the wider environmental dimension of AFR has comparatively been neglected.

## AFR in the Environment

**Figure 1:**
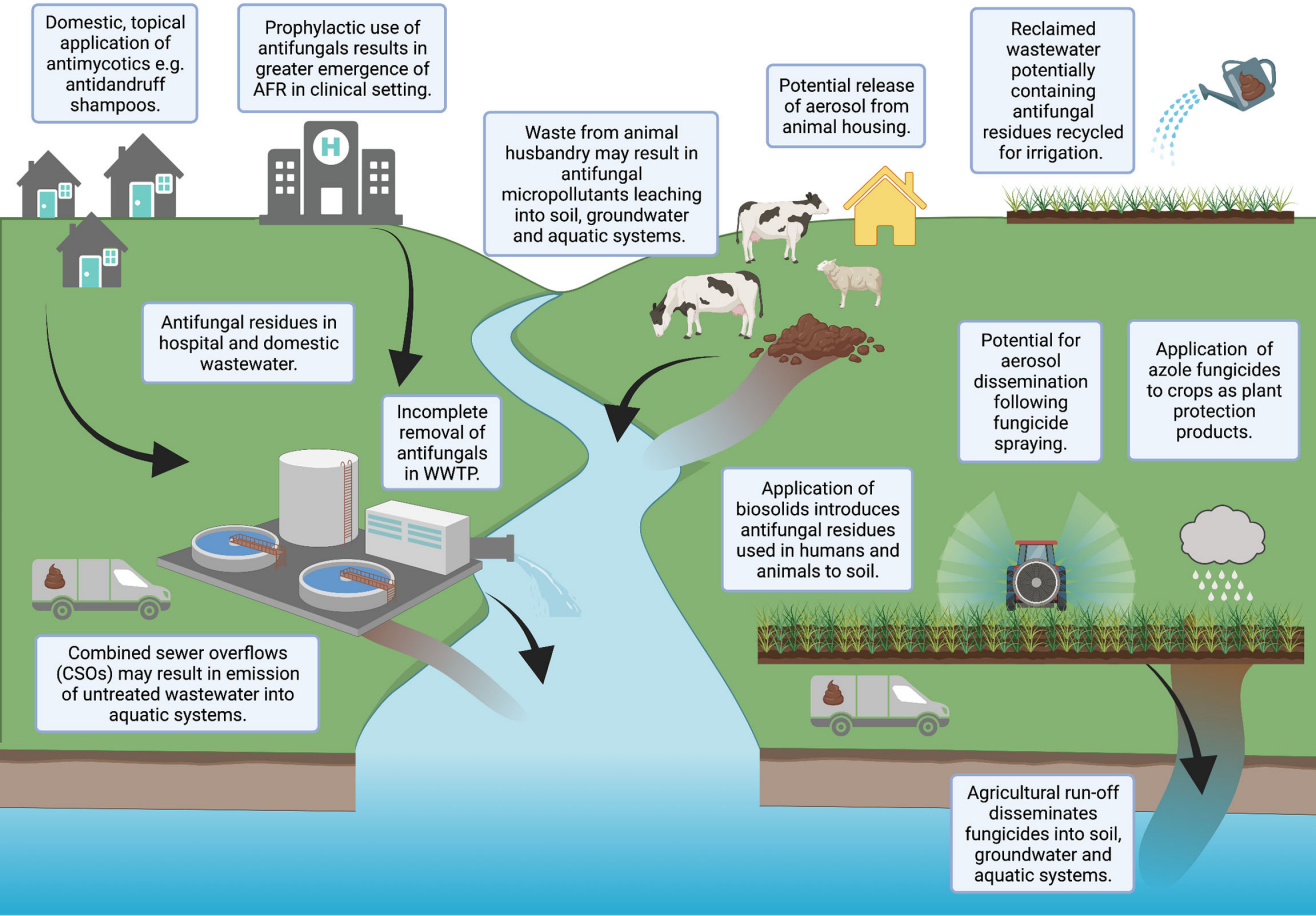
Potential pathways for antifungals to enter the environment. Created with BioRender.com.

Uses of antifungal compounds vary, including therapeutic use in human and/or veterinary medicine; use in personal care products, such as antidandruff shampoos (Richter et al., 2013); and the application of fungicides as plant protection products (PPPs) in agricultural settings. The three primary classes of antifungal agents used in the clinic are echinocandins, azoles and polyenes (Ksiezopolska and Gabaldon, 2018). The azoles are used in both human/veterinary medicine and in agriculture (Fisher et al., 2018). Given the varied applications of antifungals, there are a number of pathways for antifungals to enter the environment (**Figure 1**).

Wastewater, both domestic and clinical, has been reported to contain antifungal residues (Lindberg et al., 2010; Escher et al., 2011; Chen and Ying, 2015). Antifungals are commonly applied topically to the human body, resulting in high emissions of active ingredients (90-95%) into wastewater (Letzel et al., 2009; Peng et al., 2012; Richter et al., 2013; Assress et al., 2019; Assress et al., 2020; Assress et al., 2021b). Wastewater treatments are unable to effectively remove such compounds, resulting in antifungal contamination of sewage sludge, biosolid amended soil, wastewater effluent and receiving surface waters (Kahle et al., 2008; Peng et al., 2012; Chen et al., 2014; Assress et al., 2019; Assress et al., 2020; Assress et al., 2021b). Furthermore, reclaimed wastewater is also increasingly recycled for irrigation purposes, potentially increasing the contamination of agricultural land with antifungals not efficiently removed during treatment (Calderon-Preciado et al., 2011; Chen et al., 2011). Effluent from pharmaceutical factories (Liu and Wong, 2013; Assress et al., 2021a) and waste from industrial applications [e.g., anti-fouling paints (Andersson Trojer et al., 2013)] have also been highlighted as environmental sources of antifungals.

A further pathway for antifungals to enter the environment is the direct application of effect concentrations of azole fungicides to crops as PPPs, for instance metconazole or tebuconazole. Though most agricultural azoles are approximately 10-100 times less intrinsically active than their therapeutic counterparts (Gisi, 2014), the spraying of fungicides occurs regularly (Lago et al., 2014), with pesticide usage data indicating 1.3 million kg of triazole fungicides were applied to crops in the UK in 2016 (Garthwaite et al., 2018). Once present in natural environments, some antifungals may accumulate and persist for long periods of time (Bhagat et al., 2021).

AFR in environmental fungal communities has been documented and suggested to increase human exposure risk to AFR pathogens (Bader et al., 2015; Chen and Ying, 2015; Chowdhary and Meis, 2018; Assress et al., 2020; Jeanvoine et al., 2020; Fisher et al., 2022; Rhodes et al., 2022). However, little research has investigated the selective potential of antifungals for AFR at environmentally relevant concentrations. Without empirical data to interpret the selective risk of measured environmental concentrations (MECs) of antifungals, our ability to effectively inform safe environmental thresholds is severely limited.

## Antifungal Monitoring, Risk Assessments and Selective Concentrations

The ‘traditional selective window’ hypothesis proposes that selection for resistance will only take place at antimicrobial concentrations above the minimum inhibitory concentration (MIC) of susceptible strains, and below the MIC of resistant strains (Gullberg et al., 2011; Murray et al., 2018). However, data for ABR have challenged this assumption, revealing selection at very low, sub-clinical concentrations in both single species and complex microbial communities (Gullberg et al., 2011; Gullberg et al., 2014; Liu et al., 2015; Le Page et al., 2017; Kraupner et al., 2018; Murray et al., 2018; Murray, 2020; Murray et al., 2020; Stanton et al., 2020). Hence, it is possible that AFR selection may also take place at concentrations below the MIC of susceptible fungi, but still at environmentally relevant concentrations.

European regulations dictate that an environmental risk assessment (ERA) for a pharmaceutical is required where the predicted environmental concentration (PEC) exceeds 10ng/L (Huschek et al., 2004; Le Page et al., 2017). Traditionally, an ERA will be based on ecotoxicological tests (e.g., reproductive toxicity (Bhagat et al., 2021)) that generate a No Observed Effect Concentration (NOEC). Predicted No Effect Concentrations (PNECs) can then be generated by dividing the NOEC by an assessment factor (Murray et al., 2021).

Ecotoxicological risks have been used to inform the European Water Framework Directive’s ‘Watch List’, where compounds of concern are included if the PEC or MEC frequently exceeds the PNEC (Cortes et al., 2020). Until recently, AMR endpoints have not been considered in such assessment frameworks, although antimicrobial selective concentrations are now considered in terms of ABR selection risk. Though there are ten antifungals on the most recent iteration of the Watch List (Cortes et al., 2020) (three clinical compounds: clotrimazole, fluconazole & miconazole; and seven agricultural compounds: enilconazole, ipconazole, metconazole, penconazole, prochloraz, tebuconazole & tetraconazole), AFR selection is not an endpoint that is currently considered within antifungal ERAs (e.g., for pesticides), and it is unclear whether existing ecotoxicological assays are protective of AFR selection at environmentally relevant concentrations (Le Page et al., 2017). Importantly, previous research on antibacterial drugs, often referred to as antibiotics, has shown that conventional ecotoxicological test data using traditional endpoints [e.g., neurotoxicity and developmental toxicity (Bhagat et al., 2021)] are not always protective of ABR selection (Bengtsson-Palme and Larsson, 2016; Le Page et al., 2017; Tell et al., 2019; Murray et al., 2020), raising concerns this may also be the case for AFR.

The threshold at which AFR or ABR strains are enriched in relation to susceptible strains is defined as the minimal selective concentration (‘MSC’). Gullberg et al. (2011) first conceptualised the experimental determination of the MSC for antibacterials, using a competition-based, single-species evolution assay. This identified that the MSC for antibacterials can be > 200 times lower than the MIC of the susceptible strain (Murray et al., 2021). There have been a number of noteworthy publications since this work, which were recently reviewed by Murray et al. (2021). However, there are no previous attempts to experimentally determine MSC data for antifungals, nor is there an established definition for an antifungal MSC. This review recommends the following definition: the lowest concentration of an antifungal at which positive selection for AFR occurs.

Though there are no experimental MSC data currently available for antifungals, PNECs for resistance (PNEC^R^s) have been theoretically estimated for a number of key antifungal agents (Bengtsson-Palme and Larsson, 2016). These PNEC^R^ values were derived by dividing the lowest 1% of observed MICs obtained from the European Committee on Antimicrobial Susceptibility Testing (EUCAST) database by an assessment factor of 10, therefore accounting for the hypothesised differences between MICs and MSCs (Bengtsson-Palme and Larsson, 2016). This data has aided initial developments in assessing antifungal selective risk. Assress et al. (2021b) performed an elaborate monitoring assessment on eight azole antifungals in wastewater and surface waters in South Africa. Based on risk quotient [RQ; generated by dividing the MEC or PEC by the PNEC (Murray et al., 2021)] calculations, this work found that the MECs of antifungals did not pose a high risk to aquatic organisms (algae, daphnia and fish) using traditional ecotoxicity endpoints. However, using the PNEC^R^s generated by Bengtsson-Palme and Larsson (2016), concentrations of fluconazole and itraconazole were found ‘to pose moderate to high risk for development of AFR’ (Assress et al., 2021b). This supports the concern that ecotoxicologically derived endpoints may not always be protective of resistance selection, and thus reinforces the importance of developing experimental tools to determine empirical MSC data for antifungals.

## Commonalities and Contrasts Between Antifungal and Antibacterial Resistance

Whilst there are clear differences between bacterial and eukaryotic genetics and physiology, some elements of our understanding of ABR are likely to be transferable to AFR. Where possible, this review will compare and contrast AFR and ABR to establish experimental tools for the study of AFR, informed by methods that have previously been used to generate antibacterial MSC data.

### Mode of Action

**Figure 2:**
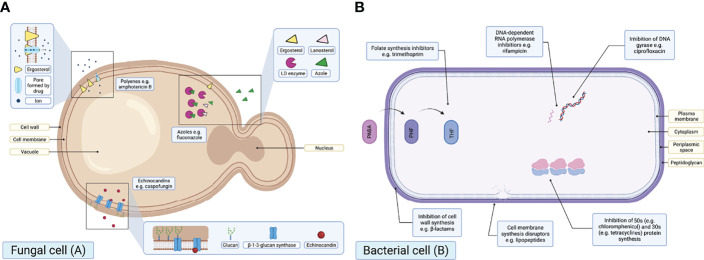
Schematic diagrams illustrating the MoAs of the primary antifungal drug classes in a fungal cell **(A)** and primary antibacterial drug classes in a bacterial cell **(B)**. PABA, para-aminobenzoic; DHF, dihydrofolic acid; THF, tetrahydrofolic acid [adapted from Sanseverino et al. (2018)]. Created with BioRender.com.

The mode of action (MoA) across the primary antifungal classes is similar (**Figure 2**) in consistently impairing cell structure and rigidity by interacting with either cell wall or cell membrane constituents. Azoles target the ergosterol biosynthesis pathway by inhibiting the enzyme lanosterol demethylase (LD), which is encoded by the *erg11* gene in yeasts and the *cyp51* gene in moulds (Morschhäuser et al., 2007; Chowdhary et al., 2014; Sagatova et al., 2015; Perlin et al., 2017). The LD enzyme is responsible for the synthesis of ergosterol, a key sterol constituent of fungal cell membranes (Odds et al., 2003; Chen and Ying, 2015; Moye-Rowley, 2020), meaning azole exposure results in ergosterol depletion (Hof, 2008). Polyenes act by binding to ergosterol, creating channels in the fungal cell membrane and killing cells by ‘allowing ions and other cellular components to escape’ (Perlin et al., 2017). Finally, echinocandin compounds inhibit the enzyme β-1-3-glucan synthase, which results in a depletion of glucans: an essential fungal cell wall component (Chaabane et al., 2019).

Similar to antifungals, some antibacterials target the bacterial cell wall or membrane. However, unlike antifungals, there are additional antibacterial intracellular targets (**Figure 2**). One concern relating to AFR is that there are few therapeutic antifungal alternative drug targets, in comparison to the broad range of antibacterial drug targets available. As a result, resistance to one or more classes of antifungals drastically reduces viable treatment options for potentially life-threatening infections.

### Mechanisms of Resistance

**Figure 3:**
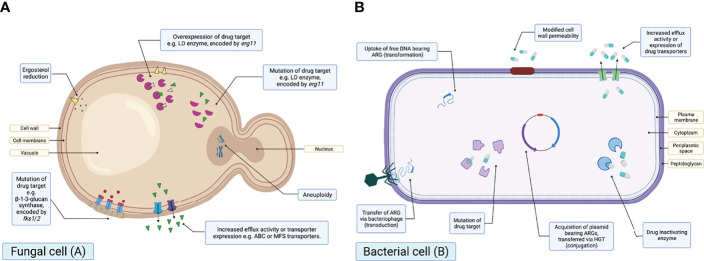
Schematic diagrams illustrating the known mechanisms of antifungal drug resistance in a fungal cell **(A)** and antibacterial resistance in a bacterial cell **(B)**. Created with BioRender.com.

When considering mechanisms of resistance, both AFR and ABR may be either intrinsic or acquired (Ben-Ami et al., 2017; Perlin et al., 2017). Intrinsic resistance includes inherent resistance to antimicrobial drugs. For instance, *Aspergillus* spp. are intrinsically resistant to fluconazole (Azevedo et al., 2015). On the other hand, acquired resistance typically follows exposure to antimicrobial selective pressures, and may be caused by mutations, genome rearrangements and/or overexpression of resistance gene products (Lockhart et al., 2017; Chaabane et al., 2019; Berman and Krysan, 2020) (**Figure 3**).

Many of the resistance mechanisms observed in fungi have also been documented in bacteria. For example, both AFR and ABR may be due to drug efflux, upregulation of resistance genes or mutations. Efflux pumps are transmembrane proteins able to actively transport drugs outside of the cell, thus reducing intracellular drug concentrations (Perlin et al., 2017; Chaabane et al., 2019; Jeanvoine et al., 2020). Overexpression involves the upregulation of genes, including *erg11*/*cyp51A* and genes encoding efflux transporters, resulting in overexpression of the proteins encoded by those genes. Finally, mutations in the amino acid sequence of drug targets can also result in resistance.

On the other hand, there are resistance mechanisms that are specific to either ABR or AFR. Mechanisms unique to AFR include changes in ergosterol synthesis and aneuploidy. For example, reduction in ergosterol synthesis *via* altered expression or mutation has been identified to result in resistance to polyenes, including amphotericin B (Young et al., 2003). Aneuploidy or aneuploids refer to cells exhibiting a chromosome number greater or less than the ‘normal’ number (Kwon-Chung and Chang, 2012), which has recently been associated with azole AFR (Sionov et al., 2010).

ABR mechanisms in bacteria may be described as either selfish or producing of ‘public goods’; referring to those that offer a community wide benefit. One of the most notably documented ABR mechanisms that produces public goods is the production of enzymes capable of degrading or inactivating antibacterials. This results in a rapid reduction in extracellular drug concentrations, allowing susceptible strains to persist in the presence of an antibacterial (Yurtsev et al., 2013; Bottery et al., 2016; Murray et al., 2018). This mechanism of ABR is widely documented, with extended spectrum beta-lactamase (ESBL) (Medaney et al., 2016) or carbapenemase producers shown to allow the growth of susceptible bacterial strains above their MIC following enzymatic degradation of antibacterial compounds (Murray et al., 2018). Fungal enzymes able to degrade antifungals have not yet been identified (Hof, 2008), therefore it could be argued that AFR mechanisms are less likely to produce ‘public goods’. Indeed, given that drug efflux is one of the predominant mechanisms of AFR and the efflux of antifungal compounds may increase extracellular drug concentrations, AFR mechanisms are more likely to be selfish than ABR mechanisms. It could also be suggested that, in comparison to bacterial communities harbouring ESBL producers, increased extracelluar antifungal concentrations resulting from drug efflux may drive further AFR evolution in fungal communities, particularly owing to the prolonged half-life of some antifungal agents (Hill et al., 2015).

Microbial communities may also become resistant to antimicrobial therapy due to phenotypic mechanisms, such as biofilm formation. As is observed with bacterial biofilm-forming phenotypes (Perlin et al., 2017), fungal biofilm communities have also been found to show increased levels of antifungal resistance. For example, *Candida* species may attach to various surfaces to form a community of sessile cells encapsuled in an extraceullar matrix (Berman and Krysan, 2020). This complex, glucan-rich matrix is able to sequester drugs e.g., fluconazole or reduce penetration of antifungal compounds, thus reducing antifungal exposure. In contrast to fungal biofilms, however, bacterial biofilms have been found to promote the horizontal gene transfer (HGT) of antibacterial resistance genes (ARGs), due to the greater proximity of individuals in the biofilm (Arias-Andres et al., 2018). 

It is key to note that conjugation, transduction and transformation are not mechanisms of resistance, but rather the three key pathways for the HGT of mobile genetic elements (MGEs) bearing ARGs (**Figure 3**). These are also unique to bacteria and there is currently no evidence to suggest this can occur within fungal communities.

### Evolution of Resistance

The longstanding assumption of AMR is that the evolutionary selective pressure i.e., antibacterial or antifungal concentration must be sufficient to offset any incurred fitness cost of resistance (Melnyk et al., 2015), and the maintenance of resistance in a population is dependent upon fitness cost in the absence of the drug (Anderson, 2005; Gagneux et al., 2006; MacLean et al., 2010; Gullberg et al., 2014; Hill et al., 2015). This implies that resistance always has a fitness cost. Generally, ABR is associated with a fitness cost (Andersson and Hughes, 2010). For example, the alteration of an antibacterial target may impair its function (Enne et al., 2005). However, fitness costs may be negated by subsequent compensatory mutations (Baquero, 2001; Enne et al., 2005; Andersson and Hughes, 2012), and resistance genes that confer a fitness advantage have also been identified (Michon et al., 2011).

Comparatively less is known about the fitness costs of AFR in fungi, though there is evidence that, as with ABR, further evolution may ameliorate incurred fitness costs *via* compensatory mutations (Cowen et al., 2002; Barker et al., 2004; Anderson, 2005; Morschhäuser et al., 2007; Hof, 2008; Sharma et al., 2015; Morschhauser, 2016; Verweij et al., 2016). In rare cases, overexpression of resistance determinants, such as efflux transporters, have led to a gain in fitness in the presence and absence of a drug (Guo et al., 2017). This is because many drug transporters are not specific to antifungals and so intracellular concentrations of other toxic compounds may also be reduced (Hof, 2008). Similarities may be drawn from this to co-selection of ABR in bacteria. Co-selection involves the indirect selection for ABR through the presence of multiple ARGs on a MGE (co-resistance) or where a single gene confers for resistance to multiple compounds (cross-resistance), for example, drug efflux. Finally, it could also be argued that fungal ploidy may have an effect on the fitness cost or maintenance of AFR in fungi. For example, a mutation on one chromosome may decrease susceptibility, whilst an unmutated gene on another chromosome may reduce associated fitness costs by retaining the original function of the gene.

There are clear differences in the evolution of resistance between fungi and bacteria (**Figure 3**). One of the most obvious differences is the absence of fungal capacity to readily take up or horizontally transfer exogenous DNA, such as plasmids (Hof, 2008; Azevedo et al., 2015). This suggests resistance development in fungal communities may be more gradual, as opposed to the ‘explosive expansion of resistance’ observed in bacteria, which may acquire multiple resistance mechanisms in one evolutionary step (Hof, 2008). Nevertheless, fungi have been described as ‘evolvable’ (Cowen et al., 2002), owing to the large number of genes encoding for resistance mechanisms (Berman and Krysan, 2020). For example, there are 30 known genes for ATP binding cassette (ABC) transporters present in the *Saccharomyces cerevisiae* genome, increasing the probability for resistance mutations to arise (Cowen, 2001; Hof, 2001). Moreover, fungal genomes (e.g., *S. cerevisiae*: 12,068kb) are significantly larger than bacterial genomes (e.g., *Escherichia coli*: 4,640kb), a factor that has been suggested to increase the likelihood of genetic mutations arising that confer AFR (Cowen et al., 2002).

## Proposed Methods to Determine AFR Selective Concentrations

**Figure 4:**
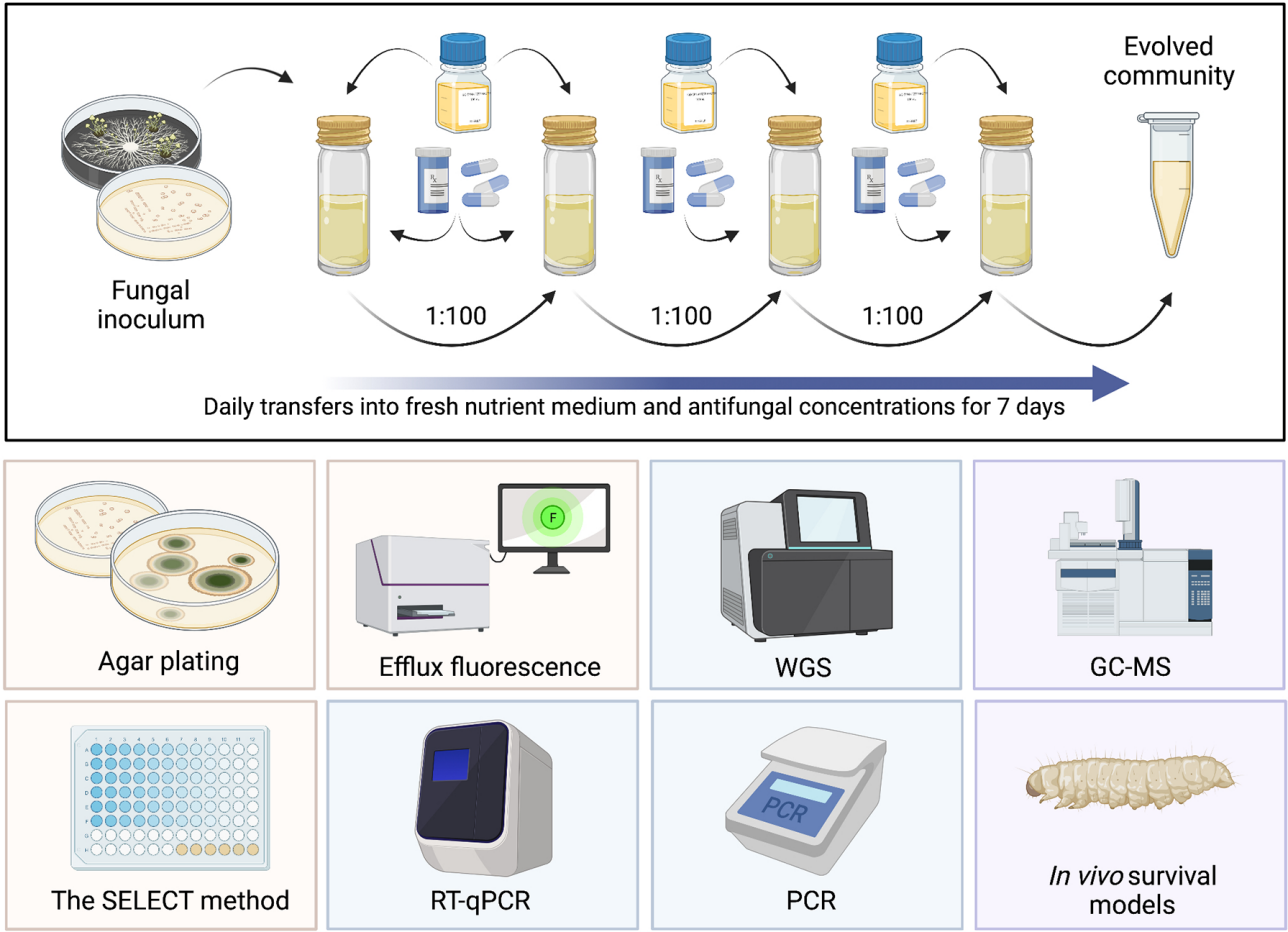
Summary of proposed assays to generate antifungal MSC data. Top: serial culture of a fungal community exposed to different antifungal concentrations for a period of 7 days, with daily transfers into fresh nutrient medium and antifungal concentrations. Yellow: phenotypic methods, blue: genotypic methods, purple: methods requiring further investigation. RT-qPCR, reverse transcription quantitative polymerase chain reaction; WGS, whole genome sequencing; PCR, polymerase chain reaction; GC-MS, gas chromatography mass spectroscopy. Created with BioRender.com.

Recent work by Murray et al. (2021) synthesised and critically appraised the methodologies previously described to determine antibacterial MSC data. From this, benefits and weaknesses in such assays were highlighted, all previous MSC data for antibacterials were collated and a novel AMR ERA framework able to identify compounds posing the greatest selection risk was proposed. Using the novel ERA framework outlined by Murray et al. (2021), antibacterial classes of high priority (in terms of generating PNEC^R^ data and assessing selection risk) were identified; namely, quinolones, cephalosporins, beta-lactams and sulphonamides. This work exemplifies the importance of having both validated methods to determine MSC data, and a streamlined framework to incorporate selection risk posed by antimicrobial compounds. To determine selection risk of environmentally relevant antifungal concentrations, experimental methods specific for antifungals must be designed and is the key focus of this section. All proposed methods are summarised in **Figure 4**.

## Phenotypic Assays

Phenotypic, culture-based assays used to determine antibacterial MSCs could potentially be modified and applied to antifungal agents. For example, Kraupner et al. (2018) established selective concentrations for ciprofloxacin (CIP) resistance in a sewage-effluent bacterial community. In summary, *E. coli* inoculated into low-nutrient medium were supplemented with and without incremental concentrations of CIP for 24 hours. Cultures were then diluted and re-inoculated with fresh medium and CIP at the same concentration after 24 hours. At 0, 24 and 48 hours aliquots of each culture were plated on chromogenic agar, supplemented with or without CIP, and colony forming units (cfu) were enumerated following incubation. Selective concentrations were defined where relative resistance (calculated by comparing cfu counts of control and CIP supplemented plates) was significantly different from the no antibacterial evolved control (Kraupner et al., 2018).

To enable application for antifungals, this method requires clear modifications, such as adoption of a fungal selective agar and specific nutrient medium (Assress et al., 2021b). In comparison with single-celled bacteria, fungal growth and morphology is more complex, particularly in moulds. It is important to note that there are key differences in the growth, structure and function of yeast cells in comparison to moulds. These differences impact the organism’s ability to evolve and maintain AFR, and therefore impact our ability to quantify resistance selection. Similarities between yeast and bacteria may be drawn upon, as they are both unicellular, undergo asexual reproduction and have typically short doubling times. This means the application of methods [including those described by Kraupner et al. (2018)] may be suited to testing antifungal selective effects against yeast cultures. Though, previous work has successfully exposed conidial suspensions of filamentous moulds, such as *A. fumigatus*, to antifungals at different concentrations before plating on selective agar (Assress et al., 2021b). This suggests there may be scope to test antifungal effects against both yeasts and moulds. Necessary modifications for moulds may include increasing agar plate incubation time, which would significantly increase the duration of the assay. For example, Assress et al. (2021b) suggest a plate incubation time of 7-10 days.

There may be opportunity to detect cross-resistance to multiple antifungal agents *via* this method. For example, 4-well plates containing minimum inhibitory breakpoint concentrations of itraconazole, posaconazole and voriconazole with an additional drug-free control have recently been developed and validated for *A. fumigatus* susceptibility testing [VIPcheck, Nijmegen, Netherlands (Arendrup et al., 2016)]. Resistance to compounds is confirmed where growth is detected in drug-containing wells. This would increase the number of compounds that could be tested and would reveal more environmentally relevant data, as environmental fungal communities are exposed to a diverse mixture of compounds in wastewater and aquatic systems. As stated, this method is currently only validated for *A. fumigatus*, however, application to yeasts may also be possible, provided appropriate antifungal breakpoints and selective media are adopted.

### The SELECT Method

**Figure 5:**
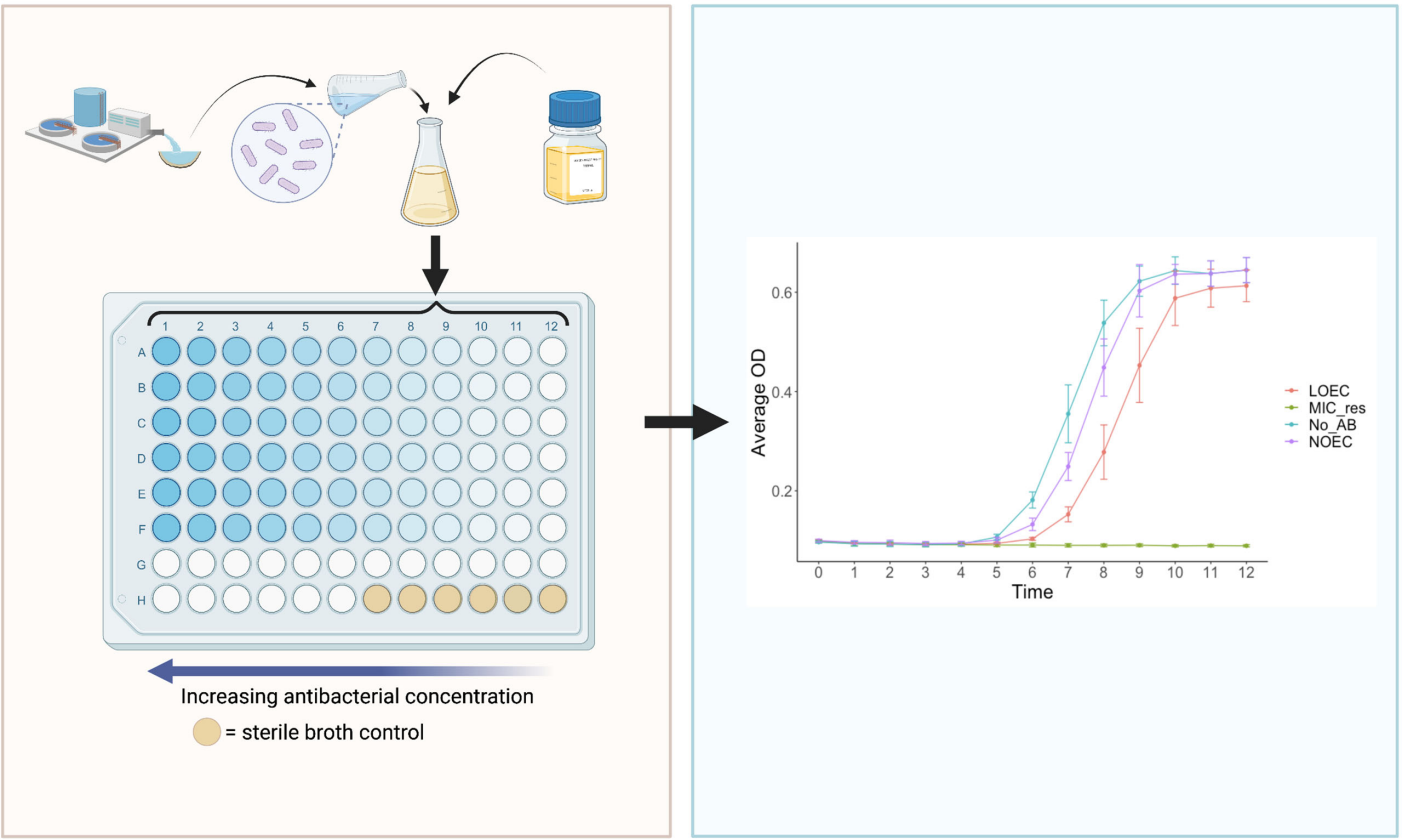
The ‘SELection End points in Communities of bacTeria’ (SELECT) Method (Murray et al., 2020). Left: a schematic overview of the SELECT assay; a 96-well-plate containing Iso-Sensitest broth inoculated with a complex sewage community, exposed to two-fold dilutions of an antibacterial. Right: an expected graphical output of the SELECT method, showing complex bacterial community growth curves in response to different concentrations of antibacterials. The LOEC is identified where the growth of the community is significantly lower than the no-antibacterial control, at the time point which exhibits the strongest dose-response relationship. LOEC, Lowest Observed Effect Concentration; NOEC, No Observed Effect Concentration; MIC_res, minimum inhibitory concentration of resistant strains, No_AB, No Antibacterial control. Created with BioRender.com.

A novel assay has recently been published that enables the rapid and cost-effective determination of antibacterial MSCs: the ‘SELection Endpoints in Communities of baCTeria’ (SELECT) method (Murray et al., 2020). In brief, the SELECT method exposes wastewater-derived complex communities of bacteria to a gradient of antibacterial concentrations (**Figure 5**). This generates selective concentrations that can be used to derive PNEC^R^s, using a significant reduction in bacterial community growth as a proxy for selection for AMR (Murray et al., 2020).

The hypothesis that community growth may be used as a proxy for ABR selection in bacteria was first proposed in the initial antibacterial MSC work from Gullberg et al. (2011). The findings from this study indicated that the MSC can be derived using selection coefficient data points to identify where the ‘numbers of resistant bacteria are expected to increase over time, relative to non-resistant bacteria’ (Murray et al., 2021). In addition, a recent mathematical model identified that a loss in net bacterial growth, especially of susceptible strains, was found to be the most sensitive for MSC predictions (Greenfield et al., 2018).

The evidence to support if reduced growth can be used as a proxy for AFR in fungi is sparse, due to a lack of experimental research. However, broth microdilution antifungal susceptibility testing (AFST) is common, and these methods follow a similar protocol to the SELECT method. For example, susceptibility profiles were previously determined across a range of *C. albicans* growth rates, measured by taking optical density (OD) measurements (Baillie and Douglas, 1998). Given the lack of further evidence, it is crucial to consider accompanying or alternative assays to empirically determine if observed growth reduction in a SELECT-type assay is associated with selection for known resistance determinants.

Initial considerations to enable use of antifungals using the SELECT method include changes to experimental inoculum and conditions. Use of a sewage derived inoculum in the original SELECT method is advantageous as the sewage microbiome is ‘representative of both the human gut, hospital effluent and WWTP influent’ (Murray, 2017). Importantly, this provides a diverse community of bacteria and resistance mechanisms for selection to act upon, including *de novo* mutations and MGEs that can be transferred between bacteria *via* HGT. For fungi, this may be less important as HGT does not occur and most AFR will be conferred by mutation or upregulation of resistance genes. Therefore, though evidence suggests fungal communities are an important component of the sewage microbiome (Whaley et al., 2016; Niu et al., 2017; Zhang et al., 2018; Chaabane et al., 2019; Bhattacharya et al., 2020), initial modifications of the SELECT method should consider focussing on a single-species yeast inoculum e.g., *Candida* spp. A weakness in restricting experimental inocula to a single-species is that this significantly reduces environmental realism (Bengtsson-Palme and Larsson, 2016; Le Page et al., 2017; Murray et al., 2020; Murray et al., 2021), as microorganisms predominantly live in complex natural communities (Van Dijck et al., 2018; Assress et al., 2019). At the same time, there are strengths in single species assays as they are less complex, allowing greater control over test organisms [e.g., ploidy (Cowen et al., 2002)] and may generate data with lower variance. One solution to improving environmental realism of a single species assay is to use strains of the sample species with variable susceptibility profiles, such as the artificial mix of *E. coli* strains previously utilised by Kraupner et al. (2021). This will account for competition between different strains and is therefore likely more representative of ‘real world scenarios’.

## Genotypic Assays

Whilst phenotypic assays are often more cost effective and provide a community-wide representation of resistance, genotypic methods have been previously employed to generate MSC data for both single species and community based bacterial assays by targeting well characterised resistance determinants. For example, the MSCs determined by the SELECT method were initially validated against a previously published, longer-term genotypic assay (Murray et al., 2018; Murray et al., 2020; Stanton et al., 2020) using quantitative polymerase chain reaction (qPCR) to quantify prevalence of key resistance genes. This method serially cultured sewage bacterial communities for a total of 7 days in the presence of different concentrations of a test antibacterial, and effect concentrations were determined where target gene prevalence was significantly greater than the no-antibacterial control. Exposing mixed bacterial communities to different antibacterials and using genotypic assays to target known resistance determinants provides an established experimental framework that may be modified to target known AFR determinants in fungi.

The two primary resistance mechanisms observed in AFR include the inhibition of target access e.g., overexpression of efflux pumps and target modification e.g., mutations (**Figure 3**).

### Overexpression

Upregulation of genes will result in overexpression of the proteins encoded by those genes e.g., efflux pumps (**Figure 3**). Selective effect concentrations of antifungals could be determined where expression levels are significantly different to basal control levels.

Methods to profile expression (including reverse transcription qPCR (RT-qPCR)), using messenger ribonucleic acid (mRNA), are extremely sensitive and have previously been used to quantify AFR determinant expression levels. For example, using RT-qPCR, Flowers et al. (2012) investigated *erg11* regulation in fluconazole-resistant versus susceptible *C. albicans* isolates, identifying that in 75% of test fluconazole-resistant isolates, *erg11* was upregulated by at least two-fold compared to unrelated susceptible strains. Efflux protein expression has also been quantified using RT-qPCR, with one study finding efflux expression levels were consistent among susceptible isolates of *Nakaseomyces glabrata*, but with a three-fold increase in fluconazole resistant isolates (Gygax et al., 2008). These methods are significantly more expensive and time consuming relative to phenotypic assays, and variable results between laboratories may be generated due to the multiple RT-qPCR enzymes and oligonucleotides commercially available (Valasek and Repa, 2005). In addition, the reliability of RT-qPCR-based measurements are dependent on normalisation, typically using an internal-control housekeeping or reference gene [reviewed in Paul et al. (2021)].

As an accompanying validation or alternative assay, there are less costly phenotypic assays available to quantify efflux pump activity. The general principle behind such assays involves the addition of fluorescent dyes to a cell suspension, allowing the kinetics of efflux to be measured. An increase in efflux pump activity is indicated where an increase in fluorescence is observed. Bhattacharya et al. (2020) evaluated *C. albicans* efflux pump activity in association with ABC and major facilitator superfamily (MFS) transporter overexpression, using the straightforward and cost-effective fluorescence assays: alanine-β-naphthylamide (Ala-Nap) and rhodamine 6G (R6G). *A. fumigatus* transporter activity can also be determined using the same protocol outlined for R6G (Nakamura et al., 2001). Following the batch microcosm experimental guidelines, MSCs can be determined where efflux activity, conferred by increased fluorescence, is significantly greater than no antifungal controls.

### Mutation

Genetic mutations play an important role in AFR and recent advancements in sequencing technologies have allowed exploration of fungal genomes, enabling the identification of important AFR genes and target site mutations (Ball et al., 2020). Vincent et al. (2013) used whole genome sequencing (WGS) to identify mutations conferring amphotericin B resistance in *C. albicans* isolates. Though more time-consuming and expensive than many of the other methods discussed, sequencing remains the ‘gold standard’ for mutation detection (Zhao et al., 2016; Paul et al., 2021) and may be used to investigate AFR selection by comparing sequencing data of control strains and evolved isogenic strains following exposure to a range of antifungal concentrations. There are also other more specific methods available to detect mutations conferring AFR. For instance, in addition to upregulation of *erg11*, mutations in this gene can confer azole resistance in yeasts. Tetra-primer-amplification refractory mutation system-PCR (T-ARMS-PCR), restriction site mutation (RSM), and high-resolution melt (HRM) analysis methods are some of the molecular tools available to determine resistance caused by *erg11* polymorphisms. Briefly, T-ARMS-PCR and HRM are used for single-nucleotide polymorphism (SNP) genotyping (Etlik et al., 2011; Caban et al., 2016). The RSM assay detects mutations using restriction enzymes and has only been adopted once to detect *erg11* mutations (Steingrimsdottir et al., 1996; Paul et al., 2021). In a recent review (Paul et al., 2021) comparing these assays, the T-ARMS-PCR and RSM approaches were deemed marginally more sensitive in discriminating resistant and susceptible isolates than HRM analysis.

Mutations to the *cyp51A* gene are the most commonly reported mechanism of azole resistance in *A. fumigatus* (Perlin et al., 2017; Ren et al., 2017), present in over 90% of clinical azole-resistant isolates (Verweij et al., 2009). This is thought to have been driven by the widespread application of azole fungicides in agriculture (Bader et al., 2015). Specifically, tandem repeats (TRs) in the *cyp51A* promoter region are frequently observed in environmental azole resistant strains (Arai et al., 2020). The most common of these include a TR sequence of 34 base pairs with L98H mutations (TR34/L98H) and a TR sequence of 46 base pairs with Y121F/T289A mutations (TR46/Y121F/T289A) (Bader et al., 2015; Arai et al., 2020). Bader et al. (2015) PCR-amplified and sequenced the *cyp51A* gene of 55 resistant *A. fumigatus* isolates and the majority of resistant isolates (80%) harboured the TR34/L98H allele, with TR46/Y121F/T289A variants the second most frequently observed. Given that these are known mutation variants, qPCR primers and probes can be used to quantify the prevalence of such variants in a sample. Again, by following similar experimental guidelines as those outlined by Murray et al. (2020), selective concentrations may be determined where mutation variant prevalence is significantly greater than no antifungal controls.

## Areas for Further Testing

The adaptation of existing experimental tools to generate antifungal MSC data is promising, however, there may also be additional assays that are able to achieve this that have not yet been tested, including the use of reduced ergosterol content or virulence as a proxy for AFR. It should be noted that these suggestions are hypothetical and would require additional testing before application.

### Ergosterol Content Quantification

Resistance to azole compounds frequently occurs due to the upregulation of *erg11* and *cyp51A* genes, resulting in changes to ergosterol concentration. Evidence recently provided by Ballard et al. (2019) suggested that azole resistance is associated with decreased ergosterol content in *A. fumigatus* fungal membranes, as measured by gas chromatography-mass spectrometry. If changes in ergosterol content can be used as a proxy for resistance selection, ergosterol quantification assays could be modified to detect selective endpoints of antifungals. Following the experimental evolution of fungal strains in the presence of difference antifungal concentrations, AFR selection may be observed at the antifungal concentration where ergosterol in the fungal cell membrane decreases significantly below the control (i.e., no antifungal present) - analogous to that conferred by a reduction in growth in the SELECT method.

### Virulence

It is postulated that acquired AFR may result in reduced virulence (Hill et al., 2015; Ben-Ami et al., 2017), although, this is still under investigation. To previously investigate this, the invertebrate host model *Galleria mellonella* was employed and a correlation between MIC and virulence (conferred by fatal outcome) was calculated (Borghi et al., 2014). This work concluded that, owing to a similar rate of killing across resistant isolates with varying susceptibility patterns of *N. glabrata*, increased resistance did not influence virulence. However, opposing evidence suggests AFR may impose an associated virulence cost (Hill et al., 2015; Ben-Ami et al., 2017).

If reduced virulence can be used as a proxy for AFR, the *G. mellonella* model could be modified to enable detection of antifungal selection endpoints. Fungal cell suspensions exposed to different antifungal concentrations could be injected into the *G. mellonella* haemocoel, and by using mortality as a measure of virulence and reduced virulence as a proxy for resistance, AFR selection could be identified where rate of killing is significantly lower than the no-antifungal control. Mini-host model assays, including *G. mellonella*, have advantages such as their cost and simplicity allowing greater replication of larvae injection (Borghi et al., 2014). In addition, this model invertebrate has been utilised for a variety of *Candida* species, including *C. albicans* (Cotter et al., 2000; Brennan et al., 2002), *C. parapsilosis* (Gago et al., 2014), *C. orthopsilosis* (Gago et al., 2014)*, C. metapsilosis* (Gago et al., 2014), *C. tropicalis* (Mesa-Arango et al., 2013) and *C. krusei* (Scorzoni et al., 2013).

## Discussion: Comparison of Proposed Methods

The methods outlined here have the potential to address the scarcity of antifungal MSC data, but there are strengths, weaknesses, opportunities and threats specific to the different methods, which are outlined in **Table 1**. There are also advantages and disadvantages that are shared across these methods, which will be outlined in this section.

**Table 1:** Strengths, weaknesses, opportunities and threats (S.W.O.T) analysis of proposed assays [adapted from Murray et al. (2021)].

Approach	Strengths	Weaknesses	Opportunities	Threats
SELECT methodology; Murray et al. (2020)	- Robust to changes in bacterial community inocula and growth conditions - Community based assay representative of a complex, mixed sample (sewage) captures competition and selection occurring for all the available resistance genes and mutations - Viable candidate for validation against OECD test guidelines - Does not limit the MSC estimation to a particular gene or gene class	- Currently restricted to antibacterials and bacterial inocula - Additional validation experiments recommended until growth as a proxy for AFR confirmed	- Potentially adaptable for other antimicrobial compounds e.g., antifungals - Can enable better investigation into the evolution of AMR by defining target selective windows - Can be applied to polyenes and echinocandins with further investigation and adaptations - Can be used to test mixtures of antimicrobials, but further validation required	- May only be applicable to yeast species, not moulds
Single species batch microcosm - culture based; Kraupner et al. (2018)	- Use of commercially available agar plates streamlines process	- Use of single species unrepresentative of environment - Plating is less sensitive than other methods	- Can allow rapid detection of cross resistance - Use of environmental strains would increase environmental relevance - Can be applied to determine MSCs for polyenes and echinocandins	- Negative culture is not sufficient to completely rule out resistance as will only reveal culturable organisms - As resistant cfu used as endpoint, drug tolerance in fungi could be an issue
Overexpression assays of specific gene targets (e.g., RT-qPCR)		- Potentially variable results due to different enzymes and oligonucleotides commercially available - Interlaboratory differences such as changes to normalisation genes'	- Can be adapted for a community-based assay representative of a complex, mixed sample (sewage)	- Will only account for resistance due to overexpression and not target site modifications e.g., mutations - Will require identification of different targets to facilitate application to echinocandins and polyenes
Mutation analysis e.g., WGS, T-ARMs-PCR, RSM and HRM	- Conventional methods well established e.g., PCR - Not limited by pre-existing knowledge of resistance determinants as able to identify novel determinants	- More time consuming and expensive - Many assays proposed are relatively novel and therefore have minimal testing history and validation	- Allows greater exploration of widely undocumented fungal genomes - Can be utilised as a precursor assay to MSC testing to identify if resistant isolates are present – albeit more expensive than simple phenotypic AFST assays - Novel qPCR primers can be designed to quantify mutation variant prevalence	- Methods including T-ARMs-PCR, RSM and HRM have only been used to detect mutations in the *erg11* gene and are not well established
Efflux activity, e.g., Ala-Nap and R6G	- Direct measure of efflux useful in rapid identification of selective concentrations - Highlights specific mechanism responsible	- Some dyes are transport-system-specific e.g. only applied to ABC transporters	- Provides efficient validation tool for overexpression or SELECT - Can be adapted for a community-based assay representative of a complex, mixed sample (sewage)	- Only detect resistance conferred by increase efflux
*In vivo* survival models, e.g., *G. mellonella*	- Used for a variety of fungal pathogens of clinical importance - Minimal ethics consideration for a host model organism - Provides data on both resistance and *in vivo* virulence	- Hypothetical: relationship between virulence and resistance not well studied - Does not provide information on resistance genes responsible	- Can be applied to polyenes and echinocandins - Can be adapted for a community-based assay representative of a complex, mixed sample (sewage)	- Based on the theory decreased virulence confers for AMR, evidence to suggest otherwise – requires further investigation
Ergosterol content quantification; Ballard et al. (2019)	- Ergosterol is universally important to all fungi, therefore this assay may be applied to a variety of species	- Hypothetical: link between decreased ergosterol content and resistance not well documented - Does not provide information on resistance genes responsible	- Can be adapted for a community-based assay representative of a complex, mixed sample (sewage)	- Based on theory (though supported that ergosterol content is a proxy for AFR - Cannot be used to determine selective effects of echinocandin resistance as ergosterol not involved in this drug class’s mode of action

SELECT, selection endpoints in communities of bacteria; OECD, Organisation for Economic Co-operation and Development; MSC, minimal selective concentration; AFR, antifungal resistance; AMR, antimicrobial resistance; cfu, colony forming units; PCR, polymerase chain reaction; RT-qPCR, reverse transcription quantitative PCR; WGS, whole genome sequencing; T-ARMS-PCR, tetra primer-amplification refractory mutation system-PCR; RSM, restriction site mutation; HRM, high-resolution melt; Ala-Nap, Alanine-β-naphthylamide; R6G, rhodamine 6G; ABC, adenosine triphosphate binding cassette.

Phenotypic assays are inexpensive, allowing the rapid generation of highly replicable data. Moreover, phenotypic methods do not require *a priori* knowledge of resistance mechanisms present and can encompass all responsible resistance determinants in the test culture, therefore providing a population-wide representation of resistance selection.

On the other hand, genotypic assays are extremely sensitive and offer accurate measures of specific resistance mechanisms. Though, genotypic assays can usually only analyse one target, with each additional target adding further time and expense, which could compromise data quality.

An overall benefit is that all of the proposed methods can be used for both yeast and mould species, except the SELECT method which would require additional testing and modifications. A key concern for all methods described, however, is that they are limited to laboratory culturable microorganisms, with evolution experiments currently conducted under optimum laboratory conditions (e.g., high temperatures and nutrients), which are not representative of the natural environment and may lead to the possible under or overestimation of MSCs. Importantly though, nutrient and temperature conditions can be modified to improve environmental realism. Further to this, there is concern of high intrinsic resistance in many fungal species, so drug-bug combinations used with these assays must be specific. Finally, the testing of single antimicrobials does not account for complex antimicrobial mixtures and co-selection, which has been found to be of importance in environmental settings.

## Conclusion

The extent of antifungal exposure in the environment and its impact on AFR is understudied, and ERA guidelines do not require assessment of antifungals in terms of their ability to drive AFR development. Due to the incomplete removal or inactivation of antifungals during wastewater treatment and the direct application of effect antifungal concentrations to agricultural crops, the environmental dimension of AFR warrants greater attention. With fungal diseases predicted to become more common due to climate change and increasing human populations, with no effective alternatives to azole fungicides currently available, it is critical to monitor and limit these impacts to secure future food security, healthcare and the global economy. Without considering resistance selection alongside traditional ecotoxicity endpoints, the risk of AFR emergence is not currently effectively assessed and novel assays are required to generate such data. Likewise, without the ability to generate and interpret MSC data for antifungals of concern, our understanding of AFR evolution, in both the clinic and the environment, is limited.

This review describes methods that could prove valuable in addressing the lack of antifungal MSC data. Following a detailed comparison of such methods, the SELECT method is highlighted as a unique and valuable tool to determine MSC values for antibacterials and is the most cost-effective and least labour-intensive experimental option to generate antifungal MSC data, should the proposed modifications allow antifungal application. An important, initial modification proposed is the adoption of a single species system, consisting of strains with varied susceptibility profiles, rather than the mixed sewage community used in the original antibacterial SELECT method. This, in combination with the other adaptations proposed, should simplify antifungal application, account for the marked differences between the evolution of antimicrobial resistance in bacteria and fungi, and considers the key differences in eubacterial genetics and growth versus eukaryotic physiology and competition. In addition, this method provides a promising tool for future adaptations, including the use of mixed compounds or other antifungal classes besides azoles. The remaining experimental assays outlined in this review offer valuable alternatives to aid the generation of novel antifungal MSC data or may also be used as validation tools to support the application of the SELECT method for antifungals and fungi. The proposed SELECT method could also be used in combination with the other methods described, whereby the SELECT method may indicate if resistance selection has taken place, accounting for all resistance mechanisms (i.e. not limited to a particular gene or gene class), then further methods could be used to investigate specific mechanisms where relevant or necessary. This work and the generation of antifungal MSC data will significantly improve our ability to inform release limits and risk assessment of antifungal use in our wider environment.

## Data Availability Statement

The original contributions presented in the study are included in the article/supplementary material. Further inquiries can be directed to the corresponding author.

## Author Contributions

ES wrote the main manuscript text with AM. ES and JU prepared the figures, and all authors reviewed them. All authors conceptualised the project, reviewed the manuscript and performed the literature validation. WG, HW and AM were responsible for project administration. WG, AH and AM led the funding acquisition. AM supervised the project. All authors contributed to the article and approved the submitted version.

## Funding

ES and AM were supported by a NERC Industrial Innovation Fellowship (NE/R01372X/1). WG was supported by NERC Knowledge Exchange Fellowships (NE/S006257/1 and NE/V019279/1). NG and AW are supported by the MRC Centre for Medical Mycology (grant MR/N006364/2). JU is funded by a BBSRC Discovery Fellowship BB/W009625/1. Funding for this work was provided by the Environment Agency through project SC200011. NG also acknowledges support from the Wellcome Trust [Senior Investigator (101873/Z/13/Z), Collaborative (200208/A/15/Z and 215599/Z/19/Z) awards.

## Conflict of Interest

The authors declare that the research was conducted in the absence of any commercial or financial relationships that could be construed as a potential conflict of interest.

## Publisher’s Note

All claims expressed in this article are solely those of the authors and do not necessarily represent those of their affiliated organizations, or those of the publisher, the editors and the reviewers. Any product that may be evaluated in this article, or claim that may be made by its manufacturer, is not guaranteed or endorsed by the publisher.
